# Signaling ethnic-national origin through names? The perception of names from an intersectional perspective

**DOI:** 10.1371/journal.pone.0270990

**Published:** 2022-08-02

**Authors:** Billie Martiniello, Pieter-Paul Verhaeghe

**Affiliations:** Department of Sociology, Vrije Universiteit Brussel (VUB), Brussels, Belgium; University of Bologna, ITALY

## Abstract

Different methodologies rely on names, by assuming that people clearly and solely perceive signals of ethnic-national origin from names. This study examines the perception of names from an intersectional perspective in a West-European context. Firstly, we analyze whether people perceive signals of ethnic-national origin in names. Secondly, we test the excludability assumption by analyzing whether names signal also other factors. Thirdly, we distinguish between homogenous and mixed names. For these purposes, we collected data on the perception of 180 names in Belgium of Belgian, Moroccan, Turkish, Polish and Congolese origin. It appears that respondents distinguish Belgian from non-Belgian names rather than perceiving a specific ethnic-national origin. Besides, people perceive signals about a person’s gender, religiosity, social class and educational level. This implies that scholars should be precautious with comparing discrimination against ethnic groups, if ethnic-national origin is only signaled through names. Moreover, the question arises as to what we are measuring exactly, since names contain complex signals.

## Introduction

The aim of this study is the analyze what signals people perceive from names. During the last decades, research has paid much attention to racism, discrimination, prejudices and implicit biases towards ethnic minorities in social, behavioral and health sciences [1–4]. Many of these studies rely on methods whereby names are a key aspect, like correspondence tests, implicit association tests and vignette studies. The use of names relies on the assumption that one the one hand, people perceive precise signals about ethnic-national origin when seeing a name (i.e. with ethnic-national origin we do not refer to beliefs about a shared history or religious, cultural or linguistic heritage, but to the migration background of a person as measured by the country of birth of either the person or his/her parents [5]. This distinction is important to make, as in certain countries like the Democratic Republic of Congo for example—considered here as one ethnic-national origin—there are different ethnic groups in terms of culture, religion or language). It is these perceived signals that is thought to lead to a different treatment.

Names can contain invisible signals, next to other visible ones, of a person’s ethnic-national origin. Depending on the macro and micro social context that is being studied, certain signals become more relevant than others [5]. Nevertheless, that does not per definition imply that all names contain clear signals of ethnic-national origin nor that people are at all times successful at perceiving these signals. This is often ignored, as names are seldomly explicitly pre-tested and are often assumed to be good signifiers. The names used in research usually stem from previous studies, databases containing popular names or birth record data [6–8]. What is yet known, however, is that the likelihood for a name to be recognized as a certain ethnic-national origin depends on the choice of the first names as well as on the specific combination with the last name [7,9]. Generally, it is more difficult to recognize the ethnic-national origin through a name when respondents only see a first [7] or a last name [10] as compared to a combination of both.

On the other hand, most scholars using names rely on the excludability assumption. This assumption implies that the research subject’s response to a name is solely based on the ethnic-national origin signaled and perceived through that name. Hence, this assumption excludes the possibility that research subject’s response is driven by other factors that a name might signal, such as the socio-economic status of, for example, the job applicant in correspondence tests [11,12]. In survey experiments the excludability assumption translates in the assumption that, when adapting an element in a survey (e.g. using different names), the information with regard to the setting of the scenario remains unchanged. Nevertheless, providing one element, like a name, can automatically evoke other feelings or beliefs [13]. This excludability assumption, however, has rarely been investigated.

The goal of this study is, therefore, threefold. Firstly, we aim to examine whether people perceive signals about ethnic-national origin through names. Previous research on the perception of names has mainly been done in the American context [6,7,9]. Because of the different migration history and social context of the U.S. and Europe, these conclusions cannot simply be extended to Europe. Europe’s large-scale migration history started after WWII, thus being more recent [14]. Additionally, the American research tradition predominantly focuses on the distinction between broad categories, mainly differentiating Whites, Hispanics, African-Americans and Asians, whereas the European tradition considers the specific country of origin (e.g. Moroccans, Poles, Congolese or Turks).

Secondly, we aim to test the excludability assumption by analyzing whether names signal also other factors next to ethnic-national origin, such as gender, religiosity, social class and educational level. We thus perform an intersectional analysis of the perception of names. For these purposes, we collected data by means of a survey in Belgium on the perception of 180 names of Belgian, Moroccan, Turkish, Polish and Congolese origin. Thirdly, we investigate how homogenous and mixed names differ in terms of perception. Mixed names are sometimes interpreted as a sign of [the willingness for] cultural assimilation or the consequence of generational effects [5,15]. Besides, the level of both perceived and objective discrimination is found to depend on whether a person’s background is visible through different signals, whereunder the name [5]. Both elements might suggest a different perception between homogenous and mixed names.

## Correspondence tests, implicit association tests and vignette studies

Correspondence tests are considered as ‘the golden standard’ to measure discriminatory behavior [11,16] and are widely used to examine labour market and rental discrimination [17–21]. Correspondence tests are field experimental techniques, in which two similar candidates with exception for their name apply for a real job vacancy or rental advertisement. Afterwards, the reactions of employers, realtors or landlords to both candidates are compared in order to uncover ethnic discrimination. In many studies, the ethnic-national origin of both candidates is signaled by means of their names (e.g. the name in top of a resume or the signature below a rental application): the ethnic majority candidate is given a typically ‘ethnic majority’ name and the ethnic minority candidate is given a characteristically ‘ethnic minority’ name [22,23].

In written applications, visible characteristics are irrelevant, thus making names the prime potential signal of ethnic-national origin: a name might be perceived as uncommon in the studied country, thus labeling the person as having a migration background [5]. Nevertheless, it is unknown whether people make a distinction between names based on the specific country of origin, or whether names are merely categorized as being of migration background or not. However, prior European research using this methodology compared the level of discrimination between ethnic minority groups without having thoroughly tested perception of the names: at most a small sample was asked to divide the names into given ethnic categories [24] or researchers had an informal discussion about the perceptions [25]. Additionally, this way of working ignores the possibility that names also signal other factors that might trigger a certain behaviour.

The same reasoning is followed in implicit association tests for measuring racial or ethnic implicit biases. This test is a chronometric procedure that quantifies the speed in which participants can associate (contrasting) concepts [26]. For example, in some studies participants could be asked to associate typically ‘black’ or ‘white’ names with positive or negative words or images. A significant difference between the ethnic categories in the speed in making these associations should indicate an implicit racial bias. Although the implicit association test is fiercely criticized [27], it is still widely used in psychology and other behavioral sciences [28]. Although scholars sometimes use pictures or other ways to signal ethnic-national origin, names are still often used. Here as well, it is assumed that names (solely) contain (clear) signals of ethnic-national origin, which triggers biases.

Also vignette studies–known as conjoint or multi-factorial survey experiments–sometimes rely on names. In these survey experiments participants have to respond to hypothetical scenarios or situations [29]. In vignettes, candidates or people could experimentally vary according to their ethnic-national origin. Although in some studies ethnic background could be explicitly mentioned [30], it can also be signaled in the written names [31,32]. When the latter is the case, researchers rely on the assumption that names signal understandably and merely ethnic-national origin, thus lacking to apply an intersectional perspective to signals carried by names.

## An intersectional perspective

A name is related to the—ascribed—social identity of a person. The paradigm of intersectionality addresses, among other elements, the complexity of social positions and identities. Crenshaw [33] distinguishes five hierarchical lines of differentiation, namely gender, ethnicity, nationality, social class and sexuality, which arise simultaneously and in interaction with one another. Based on these elements, ones’ social identity is established. Thus, a person’s identity cannot be reduced to one of these characteristics: they all influence each other [33,34]. Consequently, oppression—including discriminatory behavior—can be seen as interwoven structures based on these different aspects that constitutes ones’ social identity [35]. These hierarchical lines of differentiation form the matrix of domination: depending on where a person is situated on every hierarchical line, and thus depending on a person’s social identity, people can be treated differently. Following this intersectional perspective, discrimination, prejudices and implicit biases are not only depending on ethnic-national origin, but also on other characteristics like gender and social class. Names therefore also carry signals about gender, national, ethnic, religious, and socio-economic identity [7,8,36,37]. Thus, a majority of the hierarchal lines of differentiation are signaled through the name a person carries.

Although most correspondence tests, vignette experiments and implicit association tests focus on names solely as a signal of ethnic-national origin, the name-giving practice is found to vary by socio-economic status, ethnic-national origin, gender and religion of the parents. Over the years, the choice of names is de-traditionalized as parents try to be innovative and creative [38]. Children are given less religious or family-related names and a rise in new names over the years is measured [39]. This de-traditionalization does not directly lead to individualization, since there are still patterns that are consistent with the ethnic or socio-economic background of the family [7,38,40,41].

Moreover, an interaction is found between ethnic-national origin and socio-economic status. Since the 1970s, African-Americans and Whites in the U.S. give increasingly different first names to their children. This trend is considered as the ‘ghettoization of names’ [41], since this pattern mostly concerns African-Americans living in segregated neighborhoods and because the names signal a lower socio-economic status [40]. When respondents are asked to relate an ethnic-national origin to a name, they are more likely to perceive a name as African-American for names given by a lower than by a higher educated mother. Similarly, people are less likely to recognize a name as Hispanic as the educational level of the mother increases [9]. On the contrary, people are more likely to perceive a name as white when the name is given by a highly educated mother [7]. Besides, Black and Hispanic names are generally rated as of a lower social class than white names [42]. Also in France, Arab names are related to economic prejudice, leading parents to give French instead of Arabic names [43].

Additionally, names also contain signals about gender [7]. Pilcher [44] illustrates how the concept of ‘doing gender’ is extended in name-giving, because names categorize people by sex which relates to expected differences of conduct between males and females. Additionally, there appears to be a relation in name-giving practices between migration background and gender: ethnic minorities are more likely to give first names that are common in the host country to their daughters than sons [45,46]. This is impacted by the traditional gender roles in the country of origin and by their level of structural and identificational integration into the host country.

Religion plays a role in the latter, as Muslims more often opt for names that are common in the country of origin and the gender difference is higher as opposed to Christians [45]. However, if the ethnic minority’s country of origin and host country have the same religion, it is easier to give a first name that is common in both countries [47]. Therefore, although name-giving is detraditionalized, it might be expected that names also contain signals about religiosity. Moroccan and Turkish names generally have Islamic roots [48,49]. However, Polish names often have religious–biblical–roots too. Therefore, these names could, because of their religious roots, be perceived as more religious. However, because Christianity was predominant in Belgium, it might be that only Moroccan and Turkish names will be recognized as more religious because of their non-Christian religious roots.

Regarding name-giving among mixed couples, ethnic-national origin and socio-economic status are found to interact. The first name given to newborns is found to reflect the power distribution in the couple [36]. In a relationship between a French woman and an Arab man, for example, whether the child is going to get a French or Arab first name is dependent on the employment status of the mother. If the mother is a white-collar worker, the child is more likely to get a French name. However, the opposite holds if the mother is a blue-collar worker. When the couple consists of a French man and an Arab woman, the child will be more likely to receive a French name [36]. Although some mixed couples opt for double names to transmit the mixed identity [50], the order of the names also reflects the power distribution [48]. Additionally, the father’s ethnic-national origin and religious identity is found to have an important weight on the name-giving of their sons [46]. Therefore, some mixed couples opt for alternating the origin of the names between newborns [48]. Based on these differences in name-giving practices, we may hypothesize that people do perceive signals of ethnic-national origin through names. However, it is to be expected that also other factors are perceived, such as socio-economic status, gender or religion.

## Data & methodology

We study the perception of names from different ethnic minority groups within the West-European country of Belgium. As many other European countries, Belgium’s population is becoming increasingly super-diverse in ethnic-national origin, religious and socio-economic terms [51]. Although Belgium was initially characterized by internal migration, the end of WWII lead to the immigration of mostly labour migrants from South-Europe, but later on also from countries as Turkey and the Maghreb [14]. Additionally, there was important euro-colonial, colonial and student migration coming from Belgium’s former colony, the Democratic Republic of Congo. The migration from East- and Central European countries, like Poland, occurred mostly after the fall of the Berlin Wall and the expansion of the European Union making the free movement of persons and labour possible.

In April 2021 we conducted a survey among a sample of 990 respondents living in Flanders, the largest and Dutch-speaking part of Belgium. We restricted the sample to inhabitants of Belgian origin (= people with the Belgian nationality and whose parents were born in Belgium). The data was gathered with attention for the age, gender and educational level structure of the population. Women, people with a bachelor degree and people below 35 years were slightly overrepresented in the sample. Therefore, post-stratificational weights were calculated. We present the weighted results, but findings with or without weight do not substantially differ (available upon request).

Although we consider respondents living in Flanders, we do not expect that the results would be strongly different if we would work with a sample from Walloon Belgium for two reasons. Firstly, Flanders and Wallonia are both part of the same country, thus sharing one migration history [14]. The percentage non-Belgians is in both Flanders and Wallonia approximately 10%. The percentage inhabitants of non-Belgian background is higher in Wallonia (23.7% in 2021) as compared to Flanders (14.7% in 2021) (data accessed via statbel.be). This might eventually lead to a somehow more successful perception of names in Wallonia. Secondly, overall attitudes towards ethnic minorities do not fundamentally differ between both areas [52]. Thus, both contexts are not crucially different.

We tested the perception of 180 names of Belgian, Moroccan, Turkish, Congolese and Polish origin (see appendix). These last four groups are among the largest ethnic minority groups in Belgium [53]. In addition, we distinguish between 100 homogenous names (for all five groups) and 80 mixed names (for the four minority groups). With *homogenous names* we refer to names where both the first and last name are from the same ethnic-national origin. *Mixed names* consist of a Belgian first name and a non-Belgian last name. Finally, we further divided the names by gender.

Belgium does not publicly provide citizens’ names divided over ethnic-national origin. Thus, we constructed combinations of first and last names by using databases with the most popular female and male first names between 2010 and 2019 as well as the most common last names in 2020 (data accessed via https://statbel.fgov.be/fr/themes/population). This gives strong indications that the names used to signal Belgian origin are indeed Belgian–i.e. that those names are common in the studied country. Although traditionally there is a difference in typically Flemish and Walloon last names, with the important internal migration in Belgium [14], names that are traditionally more Flemish are also strongly present in Wallonia and vice versa. The names we use are explicitly checked to be common in both Flanders and Wallonia. The constructed names from the tested ethnic minority groups were subsequently verified by people of the same ethnic-national backgrounds.

Depending on the context that is being studied, names can contain different signals [6]. Among a Belgian sample, a name that is common in Belgium will in the majority of the cases be perceived as a Belgian name, whereas the same name might be perceived by respondents in the Netherlands as Dutch or in France as French. What is thus important for the ‘ground truth’, is that the names used to signal Belgian origin are common names in the studied country and are also perceived as such by our sample. This is the case, as 79.5% of the respondents perceive the names we use to signal a Belgian origin as such (Table 1).

**Table 1:** Mean congruence rates for gender and ethnic-national origin.

	Congruence Belgian vs. Non-Belgian	Congruence European vs. Non-European origin	Congruence specific EU origin	Congruence specific non-EU origin	Congruence gender
**Belgian**	79,5%	88,6%	-	-	93,8%
**Moroccan**	98,9%	48,2%	-	34,0%	80,1%
**Turkish**	98,7%	42,6%	-	34,5%	62,5%
**Congolese**	98,6%	42,1%	-	11,4%	70,9%
**Polish**	98,5%	61,8%	35,0%	-	90,4%
**Mixed Moroccan**	97,1%	36,3%	-	21,3%	91,2%
**Mixed Turkish**	95,7%	26,2%	-	24,5%	91,7%
**Mixed Congolese**	95,8%	36,6%	-	16,0%	92,4%
**Mixed Polish**	95,5%	64,3%	32,6%	-	92,4%

The 180 names were randomly divided over 18 versions of the survey, so that each respondent had to rate 10 names. To avoid order-effects, the order within each set of names randomly varied. The survey consisted of several question regarding the perception of ethnic-national origin. First, respondents were asked which ethnic-national origin they would assign to each name. There were seven answer categories: ‘Belgian’, ‘Another European origin’, ‘Non-European origin’, ‘Belgian + another European origin’, ‘Belgian + another non-European origin’, ‘Another European origin + non-European origin’ and ‘Don’t know’. This question provides insight in the perception of the superficial origin of names with the rude distinction between ‘European origin’ and ‘non-European origin’. Finally, if the respondent answered a category containing ‘Another European origin’ or ‘Non-European origin’, we asked to indicate which specific country of origin in open questions.

Following Gaddis [7], we created congruence variables for the respondent’s perception of the names’ ethnic-national origin. A congruence variable is dichotomous whereby we investigate whether or not the respondent perceives a name as intended: 0 stands for ‘not congruent’ and 1 for ‘congruent’ to our intended signal. Because of the complexity of the ethnic diversity within Europe, we distinguish between three types of congruency: [1] congruence with the bold difference between Belgian and non-Belgian origin. For Belgian names, answers are rated as congruent if they chose the answer option ‘Belgian’. All other answer options are rated as not congruent. For non-Belgian names and mixed names, the opposite holds: only answering ‘Belgian’ is seen as not congruent. [2] congruence for the superficial distinction between European and non-European origin. For Belgian names, we rated the option ‘Belgian’ and ‘another European origin’ as congruent. For Turkish, Moroccan and Congolese homogenous names, ‘Another non-European origin’ and for Polish homogenous names ‘Another European origin’ is seen as congruent. For the mixed names, the same principle holds, but for the options ‘Belgian + another European origin’ (or ‘another European origin’) and ‘Belgian + another non-European origin’ [3] congruence with the specific ethnic-national origin. Naming the correct country of origin (Morocco, Turkey, Congo or Poland) or a formulation referring to the country (e.g. Moroccan) was rated as congruent.

Additionally, we asked the respondents to rate the names with respect to gender, religiosity, social class and educational level. For gender, respondents were asked to choose between four categories: ‘man’, ‘woman’, ‘x’ and ‘Don’t know’. This was also translated in a congruence variable. Besides, the respondents were asked to rate their perception of religiosity, social class and educational level of each name on a 7-point Likert scale, with an additional option ‘Don’t know’. These answers were recoded into categorical variables with 4 categories, the last category being ‘Don’t know’. The perception of religiosity was recoded into [1] Not religious, [2] Neutral, [3] Religious and [4] Don’t know; social class into [1] Lower class, [2] Middle class, [3] Higher class and [4] Don’t know; and educational level into [1] Lower education, [2] Middle education, [3] Higher education and [4] Don’t know. It is possible that by asking these questions, stereotypes are activated. We cannot rule out the possibility that respondents would not perceive these signals of gender, religiosity, class and educational level when it was not explicitly asked for.

Based on these congruence and categorical variables, we first look at the descriptive statistics. Here, we pay attention to the general trends between the ethnic-national origin of the names and name type (homogenous or mixed), as well as to within-groups differences. Afterwards, we conduct multilevel binary logistic regression analyses with individual level fixed effects on the congruence variables to control for name and respondent characteristics. For the perception of religiosity, social class and educational level we also conduct multilevel binary logistic regression analyses by creating dummy’s for each category. We conduct multilevel analysis, because our data follows a cross-classified multilevel structure, given that each respondent had to rate 10 names and each set of names was rated approximately 55 times. We treat the names as level 1 (name-level) and the individuals that rated the names as level 2 (respondent-level). The variances of level 2 are significant (p<0.001) in the null model. In line with Gaddis [7], we use the following logistic regression equation on the congruence variables for ethnic-national origin and gender as well as on the dummy’s for religiosity, social class and educational level:

$$ln\left(\frac{p}{1-p}\right)=a_{n}+b_{1}X_{n}+b_{2}V_{r}$$

whereby a_n_ = intercept of level 1 (name-level); X_n_ = name variables (name type, ethnic-national origin and gender); V_r_ = respondent variables (gender, educational level and age)

In the Tables 2 and 4 we provide the odds ratios, but to increase the readability, we converted those to probabilities in the discussion of the results. We therefore use following formula:

$$Probability=\frac{odds\;ratio}{1+odds\;ratio}$$

**Table 2:** Multilevel logistic regression analysis with individual level fixed effects on the congruence variables for ethnic-national origin and gender.

	Congruence Belgian vs. non-Belgian (n = 9900)	Congruence European vs. non-European ethnic origin (n = 9900)	Congruence specific EU ethnic origin (n = 2209)	Congruence specific non-EU ethnic origin (n = 6593)	Congruence gender (n = 9900)
Intercept	7.028 (0.217)***	6.634 (0.128)***	1.024 (0.166)	0.472 (0.133)***	45.213 (0.120)***
**Name characteristics**					
Name type (ref. Homogenous name)	0.309 (0.163)***	0.476 (0.047)***	1.032 (0.095)	0.688 (0.061)***	4.938 (0.073)***
Ethnic origin (ref. Belgian)					
Moroccan	36.570 (0.219)***	0.192 (0.093)***	-	-	0.157 (0.157)***
Turkish	23.921 (0.199)***	0.128 (0.094)***	-	-	0.075 (0.153)***
Polish	26.001 (0.203)***	0.173 (0.094)***	-	-	0.340 (0.164)***
Congolese	26.321 (0.200)***	0.160 (0.093)***	-	-	0.106 (0.154)***
Gender name (ref. Man)	1.090 (0.107)	1.015 (0.044)	0.777 (0.098)	1.090 (0.061)	0.671 (0.064)***
**Respondent characteristics**					
Gender respondent (ref. Man)	1.119 (0.141)	1.064 (0.076)	0.570 (0.116)**	0.928 (0.098)	0.992 (0.102)
Educational level (ref. master degree or higher)					
Max. secondary education	0.580 (0.189)**	0.664 (0.096)***	0.474 (0.146)**	0.713 (0.124)**	0.648 (0.132)***
Bachelor degree	0.808 (0.210)	0.913 (0.105)	0.488 (0.162)*	0.933 (0.135)	0.776 (0.145)
Age (ref. +55)					
< = 34	0.708 (0.188)	0.820 (0.102)*	0.548 (0.155)	0.689 (0.131)**	1.014 (0.137)
35-54y	0.753 (0.161)	0.822 (0.085)*	0.541 (0.131)**	0.696 (0.110)***	1.005 (0.113)
-2LLR	67231.513	47800.060	10420.277	32977.682	56503.054

p<0,001***; p<0,01**; p<0,05*.

### Ethics statement

Ethical approval has been granted within the EdisTools Project to conduct questionnaires among human participants in which participation was voluntary and after informed consent. This ethical approval has been granted by the ethical commission of the Political and Social Sciences of Ghent University. The consent of participants was informed and written. The data were processed and analyzed anonymously.

## Results

### Ethnic-national origin

Table 1 presents the descriptive statistics for the mean congruence rates, which represent the percentage respondents that correctly categorized the names on the aggregated level. Looking at the congruence rates for Belgian vs. non-Belgian origin, three findings arise. Firstly, respondents are rather successful in correctly identifying whether a name is Belgian or not. Secondly, respondents are somewhat better at identifying non-Belgian than Belgian names. For non-Belgian names, the mean congruence rate varies between about 95.5% for mixed names to about 98.5% for homogenous names. This rate goes down to 79.5% for Belgian names. Thirdly, the fluctuations between ethnic-national origin and name type, as well as within one ethnic-national origin, are negligible with respect to the rude distinction between Belgian and non-Belgian origin.

**Fig 1:**
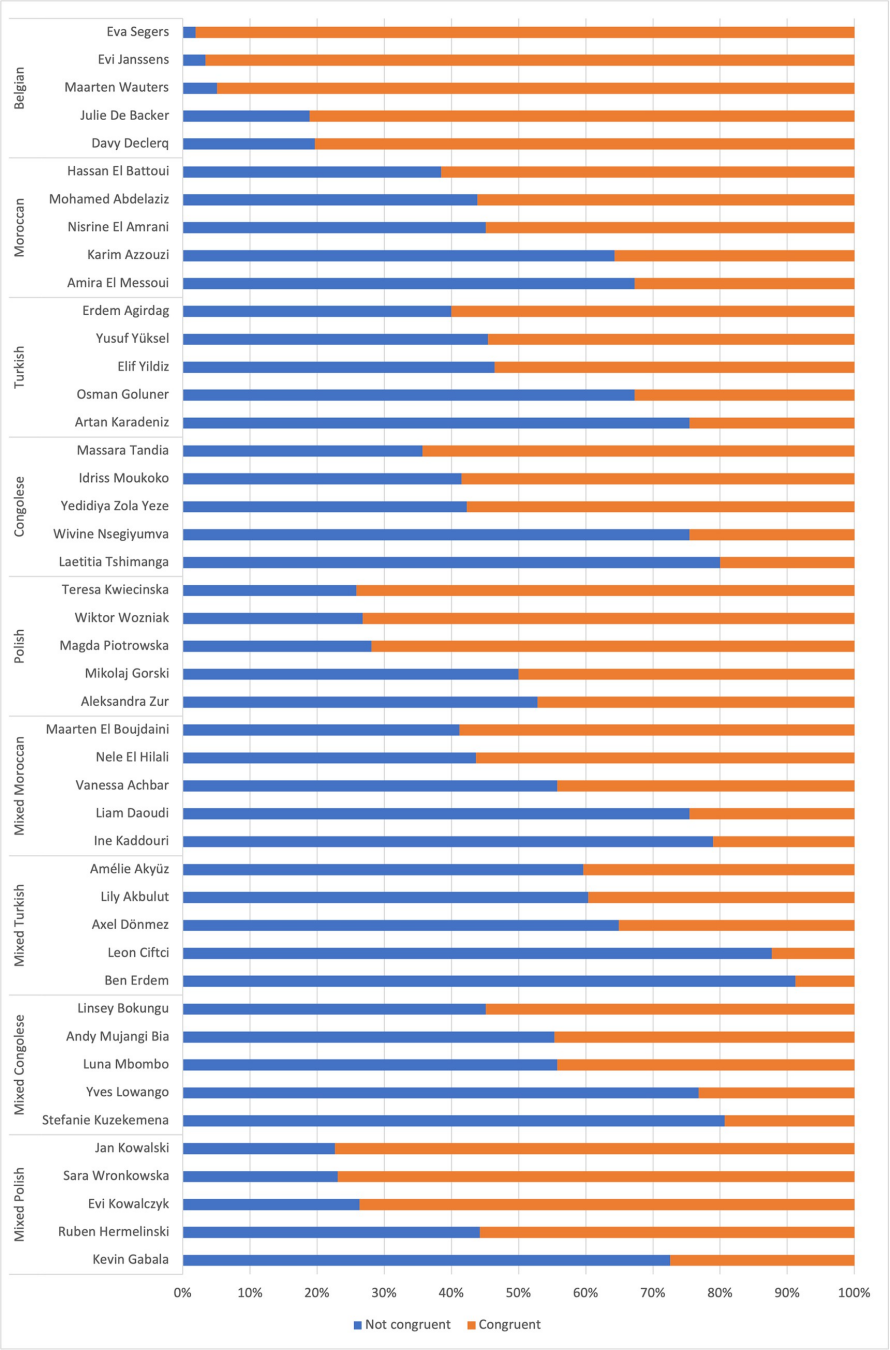


Concerning the congruence on the distinction between a European and non-European origin, 88.6% of the respondents correctly identified the Belgian names as European. As seen in Fig 1, the lowest congruence rate for Belgian names is 80.4% (Davy Declerq). When looking more closely at what other ethic national origin respondents perceive for the Belgian names, some respondents made a mistake and answered ‘Belgian’ in the open question asking to name a specific country of origin. In other cases, respondents perceived the Belgian names as Dutch or French, which are two neighboring countries with similar names as in Belgium. Only in a minority of the cases, another country of origin is cited.

The mean congruence rates for non-Belgian names (Table 1) never exceed 50%, except for Polish names, meaning that respondents are not successful at dividing the names along the distinction between ‘European’ and ‘non-European’. Additionally, respondents are more successful at identifying homogenous non-European names than mixed names. On average, 48.2% of the homogenous Moroccan names are correctly identified as non-European, followed by homogenous Turkish (42.6%) and Congolese names (42.1%). These mean congruence rates drop even more for the mixed names: the mixed Moroccan and Congolese names have almost identical mean congruence rates, with respectively 36.3% and 36.6%. Regarding mixed Turkish name, only an average of 26.2% of the names are classified correctly as a mix of European and non-European. However, respondents performed better at categorizing the Polish names as European. Here the mean congruence rate is slightly higher for the mixed (64.3%) than the homogenous (61.8%) names.

Additionally, there is important within-group variance. Fig 1 shows the three highest and two lowest congruence rates for the congruence on European versus non-European origin. The mixed Polish names have the highest fluctuations, with the highest and lowest congruence rates being respectively 77.4% (Jan Kowalski) and 27.5% (Kevin Gabała). For the mixed Turkish name, which has the lowest mean rate, the highest congruence rate is 40.4% (Amélie Akyüz) and the lowest 8.8% (Ben Erdem).

For the congruence rates on specific ethnic-national origin, respondents are even less successful. They perform less good in correctly linking a country of origin to mixed than to homogenous names for Turkish and Moroccan names. As seen in Table 1, respectively 34.5% and 34% of the Turkish and Moroccan homogenous names are correctly identified. However, when looking at the mixed names, these rates drop to respectively 24.5% and 21.3%. The rates for Congolese names are lower, with 11.4% and 16% for the homogenous and mixed names respectively. For Polish names, respectively 35.0% and 32.6% of the homogenous and mixed names are correctly identified. Nevertheless, there appears to be confusion among some respondents about which ethnic-national origin is European: both European and non-European countries are named when the question asked for a “European country” and vice versa. This is especially true for the Turkish names, which we categorized as non-European. Consequently, the percentages slightly underestimate the real congruence rates.

In Table 2 we present the results of the logistic regression analyses on the congruence rates, controlled for name and respondent characteristics. When looking at the Belgian versus non-Belgian congruence rates, respondents are 23.6% less successful at correctly identifying mixed as opposed to homogenous names. In addition, it is an easier task to identify Moroccan, Turkish, Polish and Congolese name as non-Belgian than to categorize Belgian names correctly. The gender of the rated name has no significant effect on congruence rate. Respondents with at most a secondary education degree are 36.7% less successful at the task compared to respondents with at least a master degree.

Regarding the distinction between a European and a non-European origin, the odds for congruency are 32.3% lower for mixed names as compared to homogenous names. Besides, respondents are less successful categorizing Moroccan, Turkish and Congolese names as non-European and Polish as European as opposed to Belgian names. There is again no significant difference according to the gender of the name. Besides, respondents with at most a secondary education degree and younger than 55 years old have lower odds to correctly classify the names compared to respondents with a master degree or higher or aged above 55 years old. The gender of the respondents has no influence on the congruence rates.

For the congruency on specific origin, the name type does not matter for the perception of names of European origin (here Polish names). For names of non-European origin, the odds to correctly identify the name as Moroccan, Turkish or Congolese is 40.8% lower for mixed as opposed to homogenous names. Also here, respondents with at most a secondary education degree and aged under 55 years old are less successful at to correctly perceive the origin of names as compared to respondents with a master degree or higher or aged above 55 years old. The gender of the respondents has no significant effect.

### Gender

Names appear to be a good proxy to signal gender (Table 1). 93.8% of the respondents attribute the right gender to the Belgian names. Besides, respondents more easily identify the signaled gender for mixed (approximately 92%) than for homogenous names. For the latter, respondents are most successful in perceiving the gender of Polish names (90.4%), followed by Moroccan and Congolese names (respectively 80.1% and 70.9%). Successfully attributing a gender to a Turkish name was harder (62.5%).

**Fig 2:**
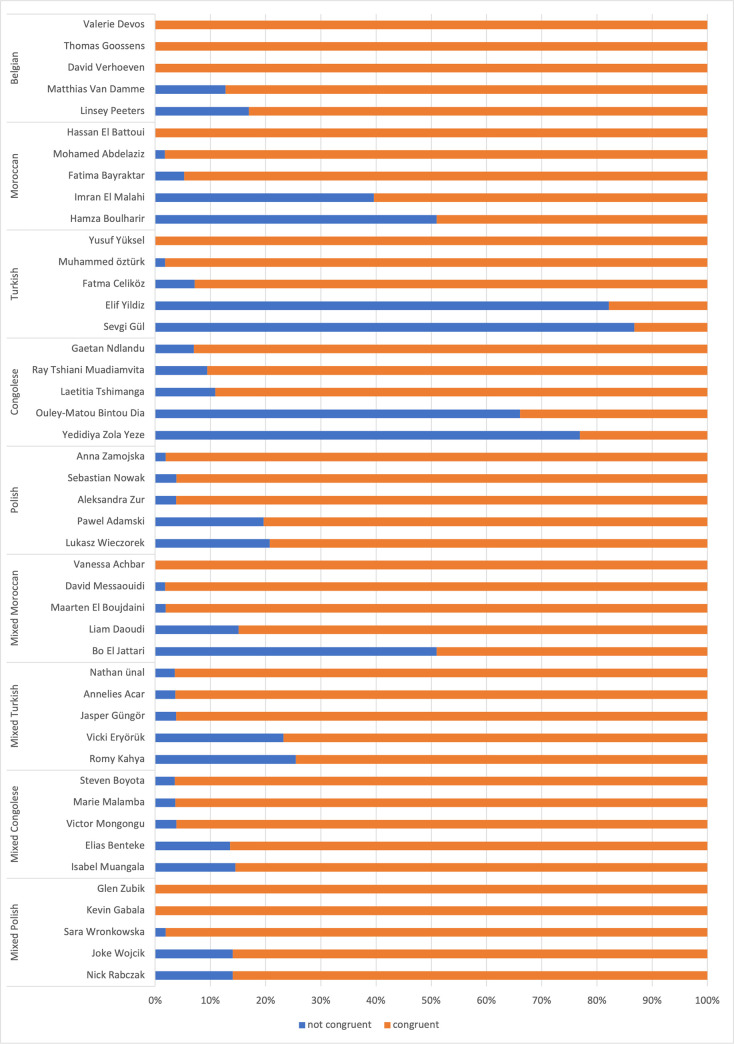


Besides, there are important within-group fluctuations for homogenous names, affecting the general rates. Fig 2 presents the three highest and two lowest congruence rates for gender. Regarding Turkish names, the lowest and highest congruence rate are respectively 13.2% (Sevgi Gül) and 100% (Yusuf Yüksel). Also for Congolese, Moroccan and Polish names fluctuations are high, with a difference of respectively 69.9%, 50.9% and 18.9%. Considering within-group fluctuations for mixed names, there is a difference of about 20% between the highest and the lowest congruence rate. Mixed Moroccan names are an exception, with 49.1% as lowest congruence rate (Bo El jattari), which might be explained by the choice of the first name. Names containing a first name that is close to a Belgian first name (f.e. Anna Zamojska, Gaetan Ndlandu) or a stereotypical first name (Mohamed Abdelaziz, Yusuf Yüksel) are more successfully interpreted.

Controlling for the name and respondent characteristics (Table 2), it appears that respondents are more successful at identifying the signaled gender for mixed names as opposed to homogenous names. The odds for congruence on gender is lower for Moroccan, Turkish, Polish and Congolese names as compared to Belgian names. In addition, respondents are less successful at recognizing female names as such as compared to male names. Regarding the characteristics of the respondents, people with at most a secondary education are 39.3% less likely to successfully perceive the gender of a name.

### Religiosity–social class–educational level

Table 3 presents the descriptive statistics for religiosity, social class and educational level. Here, there are four findings. Firstly, about 40% of the respondents answer “Don’t know” when asking which religiosity, social class or educational level they perceive from a name. Secondly, Belgian names are mainly categorized as not religious (35.1%), as middle or higher class [both 29.3%] and with a high educational level (39.1%). Contrarily, homogenous Moroccan and Turkish names are mostly perceived as religious (respectively 45.8% and 40.6%), and as lower (respectively 22.9% and 19.2%) or middle class (respectively 25.4% and 27.9%). These names are least likely to be categorized as highly educated (17.7% and 19.0% of the respondents respectively). Nevertheless, Moroccan and Turkish names are more or less equally divided over the categories of educational level. Regarding religiosity, homogenous Congolese and Polish names take up the middle positions, with respectively 35.2% and 31.1% of the respondents categorizing the names as religious. Besides, Congolese names are mostly categorized as lower (17.8%) or middle class (28.7%) and Polish names as middle class (31.4%). Additionally, both homogenous Congolese and Polish names are more often perceived as middle or highly educated.

**Table 3:** Descriptive statistics for religiosity, social class and educational level.

	**Religiosity**				**range within groups in perception as ’religious’**	
	**Not religious**	**Neutral**	**Religious**	**Don’t know**	**Lowest**	**Highest**
**Belgian**	35,1%	11,3%	11,5%	42,1%	5,8%	21,1%
**Moroccan**	3,7%	10,6%	45,8%	39,9%	33,9%	58,2%
**Turkish**	6,9%	10,8%	40,6%	41,7%	22,6%	53,6%
**Congolese**	7,5%	14,2%	35,2%	43,1%	19,6%	54,2%
**Polish**	11,8%	13,2%	31,1%	43,8%	13,2%	48,2%
**Mixed Moroccan**	11,5%	14,2%	31,3%	43,0%	12,5%	46,4%
**Mixed Turkish**	12,3%	15,2%	28,2%	44,3%	7,7%	38,5%
**Mixed Congolese**	12,5%	13,9%	29,4%	44,2%	19,3%	41,8%
**Mixed Polish**	14,2%	18,0%	25,7%	42,1%	11,3%	39,3%
	**Social Class**				**Range within group in perception as ’higher class’**	
	**Lower**	**Middle**	**Higher**	**Don’’t know**	**Lowest**	**Highest**
**Belgian**	3,4%	29,2%	29,3%	38,1%	12,3%	40,7%
**Moroccan**	22,9%	25,4%	10,5%	41,2%	5,7%	17,9%
**Turkish**	19,2%	27,9%	11,2%	41,6%	5,2%	20,0%
**Congolese**	17,8%	28,7%	12,5%	41,0%	3,7%	21,8%
**Polish**	11,9%	31,4%	14,7%	42,0%	5,0%	27,3%
**Mixed Moroccan**	12,5%	30,7%	15,6%	41,2%	5,2%	25,5%
**Mixed Turkish**	11,0%	31,3%	16,4%	41,4%	7,5%	32,8%
**Mixed Congolese**	14,8%	30,5%	13,8%	40,9%	3,9%	22,8%
**Mixed Polish**	10,5%	33,2%	15,5%	40,7%	5,9%	25,4%
	**Educational level**				**Range within group in perception as ’high educational level’**	
	**Low**	**Medium**	**High**	**Don’t know**	**Lowest**	**Highest**
**Belgian**	3,0%	15,0%	39,1%	42,9%	20,0%	50,8%
**Moroccan**	17,9%	18,5%	17,7%	46,0%	7,7%	31,0%
**Turkish**	16,4%	19,9%	19,0%	44,7%	8,6%	28,8%
**Congolese**	13,5%	20,0%	22,2%	44,3%	11,5%	38,2%
**Polish**	10,2%	19,2%	25,1%	45,5%	15,8%	34,0%
**Mixed Moroccan**	10,2%	19,3%	25,3%	45,2%	14,5%	35,1%
**Mixed Turkish**	8,2%	19,1%	26,9%	45,9%	9,4%	40,4%
**Mixed Congolese**	11,5%	19,0%	24,3%	45,2%	13,5%	38,3%
**Mixed Polish**	6,8%	19,7%	28,9%	44,6%	22,6%	37,5%

**Table 4:** Multilevel logistic regression analysis with individual level fixed effects on religiosity, social class and educational level [n = 9900].

	Religiosity				Social class				Educational level			
	Not religious	Neutral	Religious	Don’t know	Low class	Middle class	High class	Don’t know	Low education	Middle education	High education	Don’t know
Intercept	0.503 (0.200)***	0.051 (0.218)***	0.037 (0.244)***	0.562 (0.391)	0.008 (0.285)***	0.156 (0.251)***	0.248 (0.226)***	0.427 (0.425)*	0.007 (0.294)***	0.078 (0.240)***	0.410 (0.245)***	0.562 (0.428)
**Name characteristics**												
Name type (ref. Homogenous name)	2.199 (0.084)***	1.406 (0.072)***	0.465 (0.061)***	1.318 (0.100)**	0.527 (0.073)***	1.314 (0.063)***	1.452 (0.076)***	0.829 (0.121)	0.483 (0.080)***	1.000 (0.068)	1.742 (0.067)***	0.873 (0.121)
Ethnicity (ref. Belgian)												
Moroccan	0.041 (0.134)***	1.010 (0.139)	21.870 (0.135)***	0.693 (0.181)*	15.664 (0.196)***	0.837 (0.115)	0.172 (0.122)***	2.091 (0.216)***	12.212 (0.206)***	1.653 (0.131)***	0.161 (0.117)***	1.808 (0216)**
Turkish	0.060 (0.126)***	1.113 (0.138)	15.017 (0.134)***	1.012 (0.181)	11.866 (0.197)***	0.960 (0.114)	0.183 (0.121)***	2.585 (0.217)***	9.582 (0.207)***	1.764 (0.130)***	0.183 (0.116)***	1.907 (0.216)**
Polish	0.100 (0.119)***	1.449 (0.136)**	9.255 (0.135)***	0.974 (0.180)	7.324 (0.199)***	1.200 (0.114)	0.210 (0.120)***	2.620 (0.216)***	5.698 (0.210)***	1.687 (0.131)***	0.265 (0.114)***	1.898 (0.216)**
Congolese	0.067 (0.124)***	1.206 (0.136)	12.227 (0.133)***	1.321 (0.179)	13.669 (0.196)***	0.904 (0.114)	0.172 (0.121)***	2.4621 (0.215)***	9.869 (0.206)***	1.708 (0.130)***	0.178 (0.116)***	2.109 (0.216)***
Gender name (ref. Man)	0.992 (0.072)	0.992 (0.068)	1.023 (0.058)	0.997 (0.094)	0.983 (0.071)	1.095 (0.060)	0.914 (0.069)	0.973 (0.114)	1.119 (0.077)	0.931 (0.065)	1.023 (0.062)	0.907 (0.114)
**Respondent characteristics**												
Gender respondent (ref. Man)	1.021 (0.141)	1.107 (0.139)	0.745 (0.164)	1.486 (0.279)	0.790 (0.160)	1.453 (0.175)*	0.917 (0.155)	0.914 (0.297)	0.751 (0.163)	1.091 (0.160)	1.058 (0.172)	1.107 (0.300)
Educational level (ref. master degree or higher)												
Max. secondary education	1.095 (0.180)	1.183 (0.179)	1.531 (0.209)*	0.519 (0.353)	1.694 (0.212)*	1.492 (0.226)	1.725 (0.204)**	0.332 (0.378)**	1.675 (0.216)*	1.193 (0.207)	1.680 (0.224)*	0.352 (0.380)**
Bachelor degree	1.095 (0.197)	1.126 (0.196)	1.306 (0.230)	0.631 (0.388)	1.932 (0.230)**	1.440 (0.248)	1.649 (0.223)*	0.317 (0.416)**	1.736 (0.235)*	1.156 (0.226)	1.742 (0.244)*	0.385 (0.417)*
Age (ref. +55)												
< = 34	1.085 (0.187)	1.139 (0.186)	0.777 (0.220)	0.697 (0.380)	1.087 (0.211)	0.728 (0.236)	1.074 (0.205)	0.961 (0.407)	1.150 (0.215)	0.675 (0.217)	0.810 (0.233)	1.266 (0.407)
35-54y	0.758 (0.158)	1.008 (0.155)	0.509 (0.181)***	1.929 (0.307)*	0.590 (0.180)**	0.754 (0.194)	0.594 (0.174)**	2.467 (0.329)**	0.637 (0.184)*	0.699 (0.178)	0.615 (0.191)*	2.789 (0.332)**
-2LLR	59021.613	56333.308	55342.351	61679.676	59879.325	54537.212	57623.640	63513.178	61126.026	55979.618	56149.121	63274.931

p<0,001***; p<0,01**; p<0,05*.

Moroccan and Turkish names generally have Islamic roots [48,49], which might explain why these names are perceived as more religious. To control for this, we additionally consider the descriptive statistics for the distinction between biblical (7 names) and non-biblical (13 names) Belgian names (see Table II in S1 Appendix). The perception of Belgian names as religious or not does not vary according to the extent to which they have biblical roots. This suggests that Moroccan and Turkish names are perceived as more religious because of the specific religious background of those names, which differs from the historical predominance of Christianism in Belgium.

Thirdly, comparing homogenous to mixed names, the latter are perceived as less religious and more highly educated. Moroccan and Turkish mixed names more often seen as of a higher social class compared to homogenous names. However, there is no such difference between homogenous and mixed Congolese and Polish names. The difference in perception of religiosity is highest for Moroccan and Turkish names, whereby the rates differ by about 12% between homogenous and mixed names. Regarding Congolese and Polish names, the rates differ with about 5%. Although the mixed Polish names are seen as the least religious of the tested ethnic minority names (25.7%), this remains more than the double as compared to Belgian names (11.5%). Mixed names are mostly perceived as middle or highly educated whereas homogenous names are perceived as lower or middle educated. Lastly, there are within-group fluctuations.

Table 4 controls for name and respondent characteristics on the perception of religiosity, social class and educational level separately. People are 31.7% less likely to perceive mixed names as religious, 34.5% less likely to categorize mixed names as of a low social class and 32.6% less likely to rate these names as of a low educational level in comparison to homogenous names. Besides, all ethnic minority names have higher odds to be rated as religious, of a low social class and lower educational level as compared to Belgian names. Moroccan names are rated by the most respondents as religious, followed by respectively Turkish, Congolese and Polish names. Besides, Moroccan and Congolese names are most often perceived as belonging to a low social class, followed by Turkish and Polish names. Additionally, Moroccan names are most often rated as having a low rather than a middle education, followed by respectively Congolese, Turkish and Polish names. The gender of the name has no impact on the perception.

Controlling for respondent characteristics, women have 59.2% higher odds than men to perceive names as of the middle class than any of the other three categories. Respondents with at most a secondary education degree are 60.5% more likely to rate names as religious than respondents with a master degree or higher. People aged between 35 and 54 years old are 33.7% less likely to perceive a name as religious and answer “don’t know” more often than people aged above 55 years old. For the perception on social class and educational level, respondents with a secondary or a bachelor degree answer “don’t know” approximately 25% less often compared to respondents with a master degree or higher, and answer less neutrally (by choosing for low social class/educational level or high social class/educational level instead of middle class/educational level). Respondents aged between 35 and 54 years old are approximately 70% more likely to answer “don’t know” on the perception of social class and educational level than people aged above 55 years old. Besides, they are less likely to perceive names as of both a low or high social class/educational level as compared to respondents older than 55 years.

## Conclusion

This study aimed to conduct an intersectional analysis on how names are perceived. First, we examine whether the ethnic-national origin signaled through names are also perceived as such. Besides, we tested the excludability assumption, which implies that subjects solely perceive signals of ones’ ethnic-national origin through a name [12]. Moreover, we compared the perception of homogenous and mixed names. For these purposes, we conducted a survey among a sample of 990 ethnic majority respondents living in Flanders, the Northern and Dutch speaking part of Belgium, whereby we tested the perception of 180 names of Belgian, Moroccan, Turkish, Congolese and Polish origin.

There are two overarching findings. Firstly, although respondents easily attribute the signaled gender to a name, this is not the case for ethnic-national origin. Respondents appear to mainly make a rude distinction between names of Belgian versus non-Belgian origin. Perceiving a name as European or not and especially naming a specific country of origin is more difficult. Based on these results, we conclude that the intended ethnic national origin signaled through names is not always perceived as such by the average ethnic majority citizen in Belgium. Thus, not all names are a good proxy to identify a specific ethnic-national origin in Belgium. This rather low congruence differs from what Gaddis [7,9] found in the United States. There, homogenous White, Black and Hispanic names are correctly perceived by respectively 92.4% and 82.5% and 90% of the respondents. For mixed names, this congruence rate goes down to approximately 67%. The difference with our research probably lies in the context. Europe’s migration history started after WWII [14], thus being more recent. Additionally, in Europe more attention is payed to the refined ethnic-national origin (the specific country of origin) as compared to the United States where the focus often lies on the broader ethnic categories separating Whites, Hispanics, Blacks and Asians [54].

Secondly, despite approximately 40% of the respondents answering ‘don’t know’, names contain also other signals. Respondents perceive signals about gender, religiosity, social class and educational level through names. This finding contradicts the excludability assumption where correspondence tests, vignette experiments and implicit association tests rely upon and show that names contain complex signals [15,42].

These findings have two important methodological implications. Firstly, because respondents rather make a distinction between Belgian versus non-Belgian names instead of perceiving a specific ethnic-national origin, it is difficult to compare specific ethnic minority groups through only names and to draw conclusions as to which ethnic minority group is more discriminated or biased against. In other words, the external validity is affected. Some prior European research did compare the level of discrimination between ethnic minority groups, but in light of our findings this is questionable. Future correspondence tests, vignette studies and implicit association tests on ethnic discrimination and biases should take this caveat into account when they are performed within a West-European context by providing additional signals to names or by using names with a highly tested (and not assumed) congruence rate (see S1 Appendix for a few validated names).

Secondly, the question arises as to what is exactly being measured, since the excludability assumption does not hold. Here, there are two possible approaches. A first approach states that signals about religiosity, social class and educational level are related to and inseparable of the perceived signals of ethnic-national origin. More precisely, this approach sees the perception of other characteristics as stereotypes related to certain ethnic minority groups. We find indications for this approach because there are general differences between ethnic minority groups. If this is the case, we can argue that ethnic discrimination is being measured, which occurs through the existence of stereotypes. McDonough et al. [55] found indications for this approach in an American context: stereotypes related to being Hispanic, Asian or African-American, which was signaled through names on resumes, influenced the research subject’s judgements of IQ, income, writing ability and likeability. Besides, names that signal an ethnic-national origin are also found to be related to occupational stereotypes [56]. In this approach, the underlying signals about religiosity, social class and educational level in ethnic minority names might shed light on why ethnic minorities are discriminated against.

Another approach considers the signals of religiosity, social class and educational level as existing besides signals of ethnic-national origin. We also have indications for this approach, since there are important within-group fluctuations in perceptions. If this is the case, not merely ethnic discrimination is measured, but also discrimination towards one’s (perceived) religiosity, social class and/or educational level. Flage [20] illustrates discrimination towards different grounds (whereunder ethnic-national origin, gender and financial means) on the rental housing market, but the level of discrimination strongly differs between those grounds.

Based on these implications, we recommend to test the perception of names before conducting correspondence tests on the rental housing market. Regarding correspondence tests on the labour market, we recommend to signal the ethnic-national origin of candidates in more ways than just by the name (e.g. type of free time activities or language knowledge on the resume). For vignette studies and implicit association tests, we propose to also use other clues as signals, such as pictures or explicitly mentioning the ethnic-national origin, social class, educational level or religiosity. In general, we argue that, in order to increase the validity of these different methodologies, it is important to test what we are working with—here names—instead of relying on assumptions.

This research is however not without limitations. With this study, we gain insight in the perception of names based on ethnic-national origin and not based on ethnicity. However, in the Democratic Republic of Congo for example, there are different ethnic groups. Besides, the survey was conducted among a sample of adults of Belgian origin. However, research measuring ethnic discrimination using correspondence tests or implicit association tests mostly focuses on the labor or rental housing market. Thus, our research population is not the same as the research population used in those tests (e.g. real estate agents, employers, HR professionals). As the latter are usually more highly educated and older than the general adult public and have more experience, this might result in more valid perceptions of names in terms of ethnic-national origin. Future research could test the perception of names among realtors, employers and HR professionals. In addition, our results are based on the perceptions of names by adults of Belgian origin and could not be seen as representative for other West-European contexts. Nevertheless, we analyzed names from minority groups which are also important migrant groups in other West-European countries, such as the Turks in Germany, France, Switzerland and the Netherlands, the Moroccans in France, Spain and the Netherlands, the Poles in the UK, Germany and the Netherlands and the Congolese in France, Germany and the UK [57].

## Supporting information

S1 Appendix Names divided by ethnic-national origin and name type with three validated names (on gender and ethnic-national origin) in yellow. (DOCX)

S2 Appendix Descriptive statistics of biblical and non-biblical Belgian names on the perception of religiosity (n = 1.099). (DOCX)

S1 Dataset (SAV)

S1 File (PDF)
