# Supplementary material for: Signaling ethnic-national origin through names? The perception of names from an intersectional perspective
Source: PLoS One. 2022 Aug 2;17(8):e0270990. doi: 10.1371/journal.pone.0270990 (PMC9345369; doi:10.1371/journal.pone.0270990)
Supplement: S1 Appendix — (DOCX) [file pone.0270990.s001.docx]

**Appendix**

**Table I. Names divided by ethnic group and name type with three validated names (on gender and ethnicity) in yellow**

| 10 | Imran el Malahi | Fatima Bayraktar | Doruk özdemir | Ebru Gökce | Ray Tshiani Muadiamvita | Nayema Kabonogo | Tomasz Sobków | Krystyna Tabak | Davy Declercq | Evi Janssens |  | David Messaouidi | Sarah Ben Omar | Tuur Küçük | Lily Akbulut | Mathis Basenga | Sandra Nyanga | Matteo Dacyk | Kelly Mancewicz |
| --- | --- | --- | --- | --- | --- | --- | --- | --- | --- | --- | --- | --- | --- | --- | --- | --- | --- | --- | --- |
| 9 | Youssef El Ayadi | Karima El Yahyaoui | Maher Berisha Durmus | Ceylan Kiliçoglu | Radu Raileanu | Promise Semengue | Aleksander Smolarek | Gabriela Pawlak | Steven Laurent | Linsey Peeters |  | Michael Rahimi | Amy El Morabit | Davy Uzun | Zita öz | Kevin Tombolo | Debbie Lomboka | Kevin Gabała | An Ziemczyk |
| 8 | Yassin Ben Aïssa | Hanane El Yaakoubi | Yusuf Yüksel | Defne Oguz | Ouley-matou Bintou Dia | Marlene-Mae Yahuma | Łukasz Wieczorek | Zuzanna Dudek | Kevin Lemmens | Vanessa Hermans |  | Liam Daoudi | Vanessa Achahbar | Arno Turan | Amélie Akyüz | Yves Lowango | Linsey Bokungu | Nick Rabczak | Joke Wójcik |
| 7 | Rachid El Khadji | Fadua El Kaddouri | Muhammed öztürk | Meryem Aydin | Isidore Sassou-Nguesso | Maeva Bishinga | Wiktor Woźniak | Agata Zając | David Verhoeven | Cindy De Smet |  | Maxim El Moussaoui | Nina Hasani | Ben Erdem | Vicki Eryörük | Andy Mujangi Bia | Wendy Etambale | Glen Żubik | Evi Kowalczyk |
| 6 | Mohamed Abdelaziz | Norah El-Bazioui | Ahmet Karakaya | Elif Yildiz | Denzell Eden Ndiwa | Wivine Nsengiyumva | Sebastian Nowak | Aleksandra Żur | Kenny Cools | Melissa Claes |  | Jef Benthami | Lena El Makrini | Leon Ciftci | Romy Kahya | Steven Boyota | Tess Ngawa | Jurgen Calik | Natacha Koc |
| 5 | Ayoub Haddioui | Nisrine El Amrani | Artan Karadeniz | Betül Yildirim | Wilson Kaniki Masengo | Quettia Lunanga | Kacper Zawadzki | Teresa Kwiecińska | Maarten Wauters | Julie De Backer |  | Thomas Bekhalloumi | Ine Kaddouri | Nathan ünal | Liesbet çelik | Elias Benteke | Luna Mbombo | Joris Aberski | Carolien Kamińska |
| 4 | Hamza Boulharir | Soumaya El Attabi | Onur Celik | Esma Sögütlü | Yedidiya Zola Yeze | Marie-Eden Moukoko | Paweł Adamski | Anna Zamojska | Bert Vermeulen | Eva Segers |  | Maarten El Boujdaini | Bo El Jattari | Jasper Güngör | Hanne Gündüz | Daan Okito Nbombo | Marie Malamba | Jan Kowalski | Katrien Letowska |
| 3 | Hassan El Battoui | Amira El Messoui | Erdem Agirdag | Sevgi Gül | Tanguy Mangala | Laetitia Tshimanga | Rafał Kwiatkowski | Magda Piotrowska | Matthias Van Damme | valerie Devos |  | Loic El Salhi | Nele El Hilali | Axel Dönmez | Tine Gök | Victor Mongongu | Stefanie Kuzekemena | Pieter Jaworski | Sara Wronkowska |
| 2 | Youness el Malahi | Sihame Assecoum | Osman Gonuler | Fatma Celiköz | Gaetan Ndlandu | Massara Tandia | Mikołaj Górski | Dorota Dąbrowska | Pieterjan De Smet | Charlotte Michiels |  | Jacob El Majdoub | Valerie Majoui | Mattias özturk | Annelies Acar | Jules Mossemba | Juliette Bukasa | Ruben Hermeliński | Lien Ośniecka |
| 1 | Karim Azzouzi | Dounia El Majdoub | Orhan Özcan | Nimet Yilmaz | Idriss Moukoko | Eunice Makola | Henryk Borkowski | Marianna Jaśińska | Thomas Goossens | Nele Aerts |  | Tibo Akheddiou | Julie Chouirdi | Bram Yavuz | Fien Aktas | Vince Tambwe Kabati | Isabel Muangala | Simon Wyrzykowski | Hanne Gułczyńska |
| **Traditional names** | Moroccan - men | Moroccan - women | Turkish - men | Turkish - women | Congolese - men | Congolese - women | Polish - men | Polish - women | Belgian - men | Belgian - women | **Mixed names** | Moroccan - men | Moroccan - women | Turkish - men | Turkish - women | Congolese - men | Congolese - women | Polish - men | Polish - women |
