# Supplementary material for: Signaling ethnic-national origin through names? The perception of names from an intersectional perspective
Source: PLoS One. 2022 Aug 2;17(8):e0270990. doi: 10.1371/journal.pone.0270990 (PMC9345369; doi:10.1371/journal.pone.0270990)
Supplement: S2 Appendix — (DOCX) [file pone.0270990.s002.docx]

**Table II. Descriptive statistics of biblical and non-biblical Belgian names on the perception of religiosity (n=1.099)**

|  |  |  |  |  |
| --- | --- | --- | --- | --- |
|  | **Religiosity** | | | |
|  | Not religious | Neutral | Religious | Don't know |
| Not biblical | 36,4% | 10,1% | 10,4% | 43,1% |
| Biblical | 33,2% | 13,0% | 13,8% | 40,0% |
