# Supplementary material for: Signaling ethnic-national origin through names? The perception of names from an intersectional perspective
Source: PLoS One. 2022 Aug 2;17(8):e0270990. doi: 10.1371/journal.pone.0270990 (PMC9345369; doi:10.1371/journal.pone.0270990)
Supplement: S1 File — (PDF) [file pone.0270990.s004.pdf]

UNIVERSITEIT GENT

FACULTEIT POLITIEKE EN SOCIALE WETENSCHAPPEN

ETHISCHE COMMISSIE

Re: Developing training tools for explaining and reducing ethnic discrimination in the fields of education, health care, housing and labour

Principal Investigator / Researchers: Prof dr. Peter Stevens (PI), Prof dr. Eva Derous, Prof dr. Piet Van Avermaet, Prof dr. Pieter-Paul Verhaeghe Prof dr. Sara Willems and Prof dr. Stijn Baert

Dear Colleague,

The ethics committee of the Faculty of Political and Social Sciences thanks you for the thorough preparation of your application on **Developing training tools for explaining and reducing ethnic discrimination in the fields of education, health care, housing and labour.**

The committee believes that your request is a clear reflection on the ethical aspects associated with both qualitative and experimental research. Therefore the committee gives a positive advice to the application.

Yours faithfully

Prof. Dr. Sami Zemni
Voorzitter Ethische Commissie – Faculteit Politieke en Sociale Wetenschappen
Universiteitstraat 8
9000 Gent
Sami.Zemni@UGent.be
